# Artificial Intelligence in Nurse Handover: Opportunities, Risks, and Future Directions

**DOI:** 10.1002/nop2.70723

**Published:** 2026-11-05

**Authors:** Kalpana Singh, Sirwan Khalid Ahmed, Abdulqadir J. Nashwan

**Affiliations:** ^1^ Nursing & Midwifery Research Department Hamad Medical Corporation (HMC) Doha Qatar; ^2^ Department of Nursing Raparin Technical and Vocational Institute Sulaymaniyah Kurdistan Region Iraq

## 1 Introduction

Effective nurse handovers are essential for maintaining patient safety and continuity of care; however, traditional handover processes—typically verbal exchanges or handwritten notes—remain vulnerable to inefficiencies, inconsistencies, and information gaps. These methods often lack standardization, rely heavily on individual communication styles, and are prone to omissions under time pressure. (Humphries et al. 2020). Handover communication involves transferring critical information about patients' conditions, treatments, and care needs (Chalke 2014). Whether conducted verbally or through documentation, these processes are prone to errors arising from incomplete data, communication breakdowns, and variability in content, which may result in delayed treatment, duplication of tasks, medical errors, compromised patient outcomes, and increased organizational risk.

The complexity of clinical environments requires timely and accurate exchange of essential information within constrained timeframes (Tan et al. 2021), while shift‐related time pressures further increase the risk of incomplete transfers (I. Ahmed 2022).

These editorial highlights three key evidence‐informed considerations: (1) the potential of Artificial Intelligence (AI) to enhance handover efficiency and reduce cognitive burden, (2) associated risks and limitations, and (3) governance and ethical safeguards required for safe implementation.

Nurses must process large volumes of patient information within limited timeframes, contributing to cognitive overload and increasing the likelihood of missed information (Arnold et al. 2023). While structured tools such as SBAR (Situation, Background, Assessment, Recommendation) provide some consistency, workflow pressures and contextual complexity often limit their consistent application, underscoring the need for innovative solutions such as AI. (Zulkifli et al. 2023). (Law et al. 2022; Magnezi et al. 2021; Dobson 2023).

AI has emerged as a potential strategy to address these challenges through technologies such as natural language processing (NLP), machine learning, and clinical decision support systems. However, current evidence remains limited, with most applications at conceptual or pilot stages and lacking robust experimental validation. Early evidence suggests that AI may improve the organization and accessibility of clinical information during handovers (S. K. Ahmed 2023). Early evidence suggests that AI may support the organization and accessibility of clinical information during handovers, but these findings remain preliminary and require further validation.

NLP techniques can extract and summarize key data from electronic health records, potentially improving completeness of information transfer (Rathore and Rathore 2023). By highlighting clinically relevant changes—such as medication updates or abnormal vital signs—AI may support prioritization of care while complementing, rather than replacing, clinical judgement (Wehkamp et al. 2023). AI could assist prioritization of care while complementing, rather than replacing, nurses' clinical judgement. Importantly, AI implementation should be nurse‐led, locally validated, and integrated into workflows to ensure safety and relevance.

AI may also reduce variability in communication by providing structured templates and standardized checklists aligned with best practices (Akhtar et al. 2023). AI‐based clinical decision support systems can offer additional insights; however, their effectiveness in real‐time handover contexts remains under‐evaluated (Steffny et al. 2023). Furthermore, AI systems may reduce cognitive burden by filtering and presenting only relevant information (Chakladar et al. 2023; Nashwan et al. 2024), while voice recognition technologies can enhance documentation through automated transcription (Sparling et al. 2023). Despite these advantages, evidence demonstrating improvements in measurable outcomes such as reduced errors or shorter handover duration remains limited.

AI applications in practice may include automated handover summaries and risk stratification tools. For example, AI‐generated summaries can present structured patient information, while predictive models may identify patients at risk of deterioration (King et al. 2023; Vald et al. 2025). However, these applications should be interpreted cautiously given the current evidence base.

Several limitations warrant careful consideration. Over‐reliance on AI may reduce nurses' situational awareness and critical thinking, particularly when outputs are accepted without verification (King et al. 2023). The accuracy of AI systems depends on data quality; incomplete or biased data may lead to misleading outputs. Frequent alerts may also contribute to alert fatigue, reducing responsiveness to important warnings (Vald et al. 2025). Practical barriers—including system interoperability, cost, and training requirements—further constrain implementation (Blease et al. 2019).

The use of AI in nurse handovers raises important ethical and governance issues, including accountability, patient consent, data ownership, cybersecurity, and algorithmic bias. Accountability must be clearly defined, with shared responsibility among clinicians, healthcare organizations, and technology developers. While nurses remain responsible for clinical decisions, organizations must ensure that AI systems are safe, validated, and appropriately implemented. Patients may be unaware that their data are processed by AI systems, highlighting the need for transparency and informed consent (Nouis et al. 2025).

Data security and ownership require robust institutional policies, including encryption, secure storage, and controlled access. Algorithmic bias presents a significant risk, as AI systems trained on non‐representative datasets may reinforce health inequities. Regular audits, diverse datasets, and clinician oversight are essential to ensure fairness and reliability.

A phased implementation approach is recommended. Pilot testing AI tools—such as NLP‐based handover summaries within clinical settings—can help evaluate feasibility and effectiveness. Key outcomes should include handover duration, documentation completeness, error rates, and user satisfaction. Beyond clinical metrics, improvements in handover practices may enhance organizational efficiency, strengthen team coordination, and support quality and safety management frameworks. Effective handovers can reduce duplication of work, improve interdisciplinary collaboration, and contribute to a culture of accountability and patient‐centered care. Successful integration depends on adequate digital infrastructure, workforce training in AI literacy, and continuous feedback mechanisms to address usability and safety concerns.

Furthermore, the manuscript does not sufficiently address the implications for other healthcare professionals who collaborate with nurses. Doctors, allied health staff, and administrative personnel often rely on nursing handovers for continuity of care. Enhancing handover quality through AI may therefore improve interdisciplinary communication, reduce errors across teams, and foster integrated care delivery.

Future research should prioritize rigorous study designs to evaluate the impact of AI on patient safety, efficiency, and clinical outcomes. Recent work by Nouis et al. (2025).

An evidence‐informed framework linking AI functions to their benefits, risks, and required safeguards is presented in Table 1, providing a practical guide for clinicians and organizations considering implementation.

**TABLE 1:** Evidence‐informed framework for AI in nurse handovers.

AI functions	Benefits	Risks	Required safeguards	Example use case	Outcomes to monitor
NLP summarization of EHRs	Improved data transfer	Misinterpretation	Data validation	Automated patient summary	Completeness of documentation
Structured templates	Standardized	Overreliance	Nurse oversight	SBAR‐based handover checklist	Consistency of information
Decision support	Evidence‐informed insights	Alert fatigue	Threshold tuning	Risk stratification alerts	Response to alerts
Voice automation	Efficient documentation	Privacy risks	Secure system	Automated transcription of handover	Accuracy of records
Risk flagging	Early detection	False alert	clinical validation	Predictive deterioration alerts	Patient safety outcomes

*Note:* EHR = Electronic Health Record; NLP = Natural Language Processing.

## 2 Implications for Nursing

- AI can support nurse handovers by improving information organization, reducing cognitive burden, and enhancing documentation.
- Implementation should be cautious, with strong emphasis on data quality, validation, and clinician oversight.
- Nurses must remain actively engaged in clinical decision‐making to avoid over‐reliance on AI systems.
- Healthcare organizations should invest in training, infrastructure, and governance frameworks to ensure safe adoption.
- Ongoing evaluation using measurable outcomes (e.g., errors, efficiency, user satisfaction) is essential.

## 3 Implications for Practice

AI has the potential to support nurse handovers by improving information organization, reducing cognitive burden, and enhancing documentation. Nurse involvement is critical in the design, testing, validation, and monitoring of AI tools. Nurses should verify AI‐generated summaries, receive AI literacy training, and ensure integration into local workflows. Ongoing audits for safety, bias, and usability are essential to safeguard patient care.

## 4 Conclusion

AI represents a promising but evolving tool for enhancing nurse handovers. Its role should be viewed as augmenting rather than replacing clinical judgement. Given the current limitations in high‐quality evidence, cautious, evidence‐based implementation is essential. Future research should prioritize rigorous study designs to evaluate the impact of AI on patient safety, efficiency, and clinical outcomes. AI tools may support, but should not substitute, nurses' expertise. Nurse‐led, locally validated approaches are key to safe adoption.

## Author Contributions

**Sirwan Khalid Ahmed:** writing – review and editing. **Kalpana Singh:** conceptualization, methodology, formal analysis, writing – original draft, writing – review and editing. **Abdulqadir J. Nashwan:** writing – review and editing.

## Funding

The authors have nothing to report.

### Conflicts of Interest

The authors declare no conflicts of interest.

## Data Availability

Data sharing not applicable to this article as no datasets were generated or analysed during the current study.
